# Reduction in Errors in Roughness Evaluation with an Accurate Definition of the S-L Surface

**DOI:** 10.3390/ma16051865

**Published:** 2023-02-24

**Authors:** Przemysław Podulka, Wojciech Macek, Ricardo Branco, Reza Masoudi Nejad

**Affiliations:** 1 Faculty of Mechanical Engineering and Aeronautics, Rzeszow University of Technology, Powstancow Warszawy 8 Street, 35-959 Rzeszów, Poland; 2 Faculty of Mechanical Engineering and Ship Technology, Gdańsk University of Technology, Narutowicza 11/12 Street, 80-233 Gdańsk, Poland; 3 Department of Mechanical Engineering, Centre for Mechanical Engineering, Materials and Processes (CEMMPRE), University of Coimbra, 3030-788 Coimbra, Portugal; 4 School of Mechanical and Electrical Engineering, University of Electronic Science and Technology of China, Chengdu 611731, China

**Keywords:** surface topography, surface texture, roughness, S-L surface, form removal, measurement noise

## Abstract

Characterization of surface topography, roughly divided into measurement and data analysis, can be valuable in the process of validation of the tribological performance of machined parts. Surface topography, especially the roughness, can respond straightly to the machining process and, in some cases, is defined as a fingerprint of the manufacturing. When considering the high precision of surface topography studies, the definition of both S-surface and L-surface can drive many errors that influence the analysis of the accuracy of the manufacturing process. Even if precise measuring equipment (device and method) is provided but received data are processed erroneously, the precision is still lost. From that matter, the precise definition of the S-L surface can be valuable in the roughness evaluation allowing a reduction in the rejection of properly made parts. In this paper, it was proposed how to select an appropriate procedure for the removal of the L- and S- components from the raw measured data. Various types of surface topographies were considered, e.g., plateau-honed (some with burnished oil pockets), turned, milled, ground, laser-textured, ceramic, composite, and, generally, isotropic. They were measured with different (stylus and optical) methods, respectively, and parameters from the ISO 25178 standard were also taken into consideration. It was found that commonly used and available commercial software methods can be valuable and especially helpful in the precise definition of the S-L surface; respectively, its usage requires an appropriate response (knowledge) from the users.

## 1. Introduction

Characterization of surface topography in the manufacturing process can be valuable in the analysis of the tribological performance of machined parts. Much valuable information can be received straightly from the analysis of surface roughness data, such as wear resistance [1], lubricant retention [2], friction [3], fatigue [4,5,6], sealing [7], analysis of energy consumption [8], eco-friendly strategies [9,10], or, generally, functional performance [11,12]. In many cases, surface topography is perceived as a fingerprint of the manufacturing process [13]. When considering the precision of surface topography studies, there must be validation of both the measurement and the data processes. Errors that occur when both operations are provided can cause an erroneous estimation of properties of properly manufactured parts leading to their classification as a lack and, unfortunately, their rejection [14]. Many types of errors can be found in surface topography studies. Roughly, they can be divided into those reflected in the measurement process [15,16,17] and those connected with the whole data analysis actions [18].

It was found in previous studies that even when a highly precise measurement technique is used, if the process of data calculation and evaluation is selected erroneously, the whole surface roughness analysis is not provided appropriately. The biggest errors in data processing can result in the biggest distortion in the whole surface topography analysis [19]. Considering errors in the field of data evaluation, especially the calculation of surface roughness parameters, errors in defining an appropriate reference plane are very often encountered. From the definition, a reference plane is a plane according to which the surface topography (roughness) parameters are calculated. From that matter, when defining this plane with unappropriated methods or, respectively, by inappropriate application of proper procedures, surface roughness parameters can be falsely estimated. Thus, errors can arise in the validation of the manufactured product [20].

Generally, the surface data, especially those associated with surface topography, can be roughly divided into form, waviness, and roughness [21]. Surface roughness parameters of machined parts are calculated after areal form removal, where form includes form and waviness components. These components of surface data are defined as L-components and are included in the L-surface [22]. Distortion in a proper definition of L-surface can be especially visible when analyzing surfaces containing deep or wide features, such as oil pockets or, generally, dimples [23,24,25]. It was found that texturing of the surface, especially when creating additional oil pockets by burnishing techniques, can significantly improve the properties of the machined surface [26,27,28]. From that point of view, precise characterization of multi-process surfaces [29,30] is of great importance.

The reduction in errors in the feature characterization is another encouraging task to be resolved [31]. In general, each of the actions provided on the surface topography data, including those with the feature-based characterization, is provided for a more direct relationship between characterization, manufacturing process, and surface function [32]. The effect of feature size, density, and distribution was found crucial in the validation of methods for both an areal form removal and high-frequency measurement noise reduction [33]. The measurement noise is a type of error, simplifying, added to the output signal, which occurs when the measuring instrument (e.g., roughness profilometer) is used [34]. The measurement noise can be analyzed in various domains; the bandwidth characterization was proposed previously [35]. One of the types of measurement noise is in the high-frequency domain [36,37].

The measurement noise, described in the high-frequency domain, can be derived from the instability of the mechanics received by influences from the environment. Nevertheless, in most cases, the high-frequency measurement noise outcome from the vibration [38]. Those components of the surface data are reflected in the S-components, and the surface including those errors can be defined as the S-surface or, respectively, as the noise surface [39]. Usually, the high-frequency spatial components are eliminated by the S-filter [40,41], which removes small-scale lateral basics from the surface [22]. Small-scale (S-) components can also be removed by the proposal of the nesting index [42] term; it is an extension of the concept of cut-off. It was proposed to be the S-nesting index value and should be proposed at a 3:1 ratio with the maximum sampling distance [43,44,45]. From that matter, the L-nesting index [46] can be defined for removing the large-scale components (such as form and waviness) and, correspondingly, the S-nesting index for removing the small-scale components (e.g., high-frequency measurement errors) from the surface data. However, the process of selection of the S-filter and L-filter nesting indexes must be studied for each type of surface separately with consideration of distortion of the surface topography features. For example, the surface texture of the ink-jet printed THV films was investigated after the application of an S-filter with a bandwidth (nesting index) equal to 2.5 μm and an L-filter with a cut-off (nesting index) of 250 μm to remove high-spatial frequency noise and, respectively, long-scale waviness/form from the raw measured data [47].

Generally, when calculating the roughness parameters, both components (L- and S-) must be removed from the raw measured data. In the result, the received roughness surface, after L-filtering and S-filtering, is derived as the S-L surface [48]. Reducing errors in the calculation of the S-L surface simultaneously influences the errors in roughness evaluation. There are many papers considering the selection of methods (S-filters and L-filters) for roughness evaluation; nevertheless, errors in the false estimation of the S-L surface were not comprehensively studied. Moreover, even if the selection of the S-L surface depends on the type of detail considered (machining process and its parameters), the requirements from the analysis provided in this paper increase. In this paper, a selection of proper procedures for the definition of the S-L surface of different topographies is presented, especially with an indication of the distortion of selected features, such as dimples, scratches, and valleys. It is also considered that the reduction in errors in roughness evaluation has a straight impact on the values of ISO 25178 parameters.

## 2. Materials and Methods

### 2.1. Analysed Details

**Figure 1:**
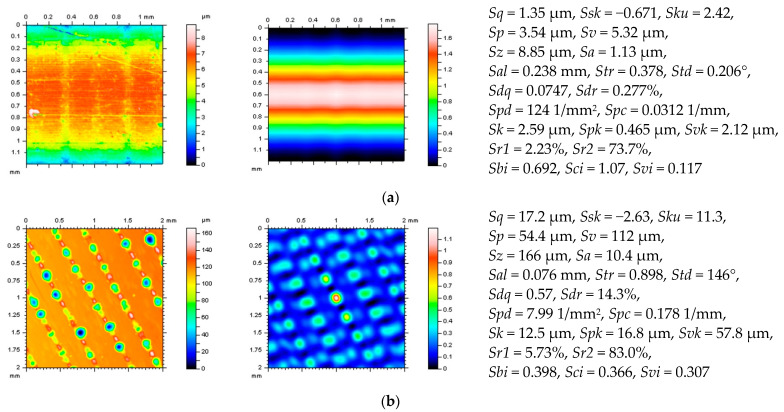

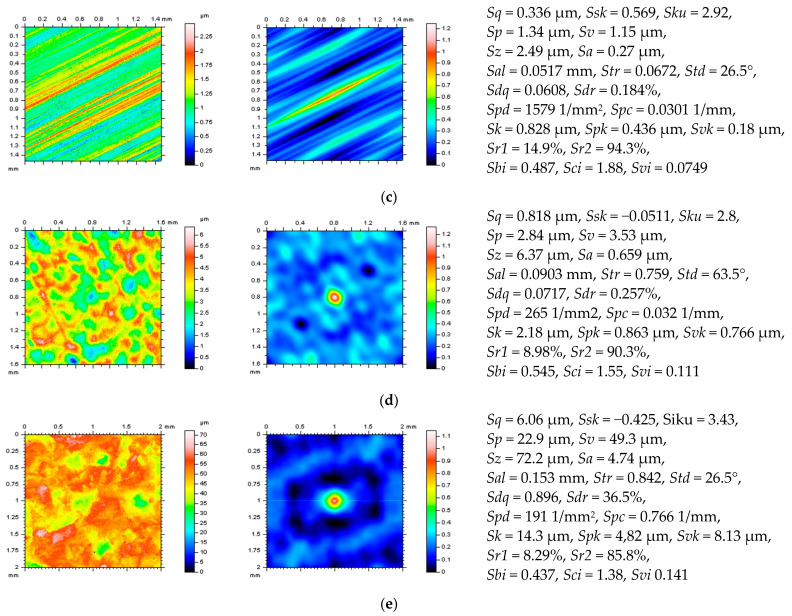
Contour map plots (left column), the 3D ACF (middle column) and ISO 25178 surface topography parameters (right column) of turned (**a**), laser−textured (**b**), ground (**c**), ceramic (**d**), and composite (**e**) surface.

Various types of surface topographies were considered, as follows: deterministic one-process turned piston skirts, ground (speed of 28 m/s, in-feed of 10 m/min, cross-feed of 1 mm/pitch, and depth of 0.02 mm), milled (depth of machining of 0.4 mm, speed of multi-cut head of 140 rev/min, and feeds of 0.3), laser-textured (with different angles of texturing, 30°, 60°, 90°, and 120°), composite, ceramic and, respectively, generally isotropic. More than 10 surfaces from each type of topography were measured and studied. Further, some of them were examined and presented in more detail. Additionally, the analyses were improved by incorporating modeled data. Then, the data were compared to the measured results in order to make general recommendations. Figure 1 shows examples of each type of surface (turned (**a**), laser-textured (**b**), ground (**c**), ceramic (**d**), and composite (**e**)) with contour map plots (left column), areal autocorrelation functions (middle column) and selected ISO 25178 surface roughness parameters (right column).

The following ISO 25178 roughness parameters from various groups were measured and studied: root-mean-square height *Sq*, skewness *Ssk*, kurtosis *Sku*, maximum peak height *Sp*, maximum valley depth *Sv*, the maximum height of surface *Sz*, arithmetic mean height *Sa* from amplitude parameters; areal material ratio *Smr*, inverse areal material ratio *Smc*, extreme peak height *Sxp* from functional parameters; auto-correlation length *Sal*, texture parameter *Str*, texture direction *Std* from spatial parameters; root-mean-square gradient *Sdq* and developed interfacial areal ratio *Sdr* from hybrid parameters; peak density *Spd* and arithmetic mean peak curvature *Spc* from feature parameters; surface bearing index *Sbi*, core fluid retention index *Sci* and valley fluid retention index *Svi* from functional indices; and core roughness depth *Sk*, reduced summit height *Spk*, reduced valley depth *Svk*, upper bearing area *Sr1*, and lower bearing area *Sr2* from the *Sk* family parameters.

### 2.2. Measurement Process

All of the analyzed surfaces were measured with contact (stylus) and non-contact (optical) methods to improve proposals for different measurement techniques.

The contact technique was based on a Talyscan 150 stylus instrument (Taylor Hobson, Warrenville, IL, USA), equipped with a nominal tip radius of 2 μm, approximately, a height resolution equal to 10 nm, a measured area of 5 by 5 mm (1000 × 1000 measured points), the sampling interval 5 μm, and the measurement speed 0.75 mm/s.

The non-contact measurement device was the white light interferometer Talysurf CCI Lite (produced by Taylor Hobson Ltd., Leicester, U.K., version 2.8.2.95), employed with a height resolution of 0.01 nm, a measured area of 3.35 by 3.35 mm (1024 × 1024 measured points), and a spacing of 3.27 μm. A Nikon 5×/0.13 TI objective was utilized.

For both analyses, areal digital filters from the TalyMap Gold (Digital Surf) software were employed to receive the ISO 25178 roughness parameters. Moreover, all of the functions proposed and validated in this paper were used from this source as well.

### 2.3. Applied Methods

For the characterization of surface topography with evaluation (calculation) of the roughness, both data analysis methods (definition of S-surface and L-surface) must be provided with error minimization. In this proposal, selected functions available in commonly used commercial software were utilized.

Often applied for the characterization of surface topography is an Autocorrelation Function (ACF). This function is described by the ISO standards and many research items [49]. In many primary studies, the ACF was proposed for the analysis of roughness isotropy [50] or anisotropy [51], description and realizations of homogeneous and isotropic two-dimensional Gaussian random processes [52], isotropic exponential and transformed exponential multiscale correlations [53], measurements of the variance of surface height obtained in several scattering geometries and also for stylus measurements [54], statistical computations of the root mean square (RMS) height, skewness (Ssk), and kurtosis (Sku) of the roughness height distribution [55], direction parallel and perpendicular to grooves, classification of data (signal) to the individual groups [56], identification of the periodicity and randomness [57], angular distribution characterization [58], the relationship between the height of a one-dimensionally rough surface and the intensity distribution of the light scattered by surface [59], vertical and lateral information about surface roughness [60], statistical irregularities of the waveguide substrate [61], characterization of the random component of the surface profile [62], determination of the domination in the frequency spectrum [63], and, frequently, characterization of the roughness measurement of the machined surfaces [64].

The ACF can also be valuable in the modeling of surface data [65], such as generating non-Gaussian surfaces with specified standard deviation [66], modeling bidirectional soil surfaces [67], influencing surface-induced resistivity of gold films [68], scale-dependent roughness modeling [69] or horizontal components [70], random generation of rough surface [71], simulations in ultrasonic assisted magnetic abrasive finishing [72], or in electro-discharge machining [73] processes when interrelationship between surface texture parameters and process parameters are emphasized. The ACF can also be valuable in the analysis of similarity and fractality in the modeling of roughness [74]. In summary, the ACF describes the dependence of the values of the data at one position on the values at another position [75].

When measuring the surface roughness, the scan resolution of scanning probe microscopy must be considered [76]. It was found that the shape of the ACF is sensitive to the measurement resolution [77]. It was also found that for very smooth surfaces, e.g., rolled or harrowed fields, the fractal process can determine mainly the overall shape of the ACF. Continuing, when considering very rough surfaces, the shape of the ACF can be determined by the single-scale process as well [78]. The ACF shape at various spatial scales, with RMS height and correlation length statistics, can be crucial in the analysis of roughness properties of the different tillage classes [79]. The ACF shape has a strong influence on the backscatter simulation results [80] as well. The influence of profile length on both the roughness parameters and the ACF shape was studied. It was assumed that the values of roughness parameters increase asymptotically with the increasing profile length [81]. The profile line shapes of the ACF intensities are obtained at different heights, and it is shown that the shapes are affected by noise [82], depending on the frequency type of noise [83]. By studying the shape of the ACF, the detection and reduction in selected types (frequencies) of measurement errors (noise) can be significantly improved [84].

Similarly to the ACF, also often applied in surface topography characterization, is the spectral analysis that can be a reliable indicator of roughness [85]. A typical example of this type of study is the application of the power spectral density (PSD) [86]. It was presented in the surface analysis that the RMS roughness depends on the length scale used for the measurement so, correspondingly, the RMS value of surface roughness is not a scale-invariant quantity [87]. From that matter, a precise description of surface morphology requires more sophisticated tools, and the PSD is classified as such a method [88]. Moreover, the PSD can be a preferred quantity when specifying surface roughness, especially considering the draft international drawing standard for surface texture [89].

Compared to the ACF, the PSD can support the modeling of surface roughness [90] as well. Profile generation [91] is received with an application of Fourier transformation. In the modeling of the surface roughness of thin films, the PSD was proposed through selected correlation [92]. Improvements in Fourier techniques to characterize the wavefront of optical components can also be received through the usage of PSD [93], especially when considering morphological parameters [94]. The vertical and lateral information of the surface profile can also be obtained with PSD applications [95]. Combining PSD with other methods, the profile roughness can be characterized by the PSD curve first and then by formation mechanisms of different frequency regions analyzed in more detail [96]. The PSD distribution can be used to explain the influence of tool feed, spindle speed, and, respectively, material-induced vibrations on surface roughness [97]. Generally, the PSD characterization can give a full description of the spatial frequency spectrum present on the surface, which is a result of interactions between all the machining parameters [98].

Both methods, ACF and PSD, were proposed and can be applied. In some cases, the applications must be provided simultaneously [99], especially when defining an appropriate L- or S-surface.

#### 2.3.1. Supporting an Areal Form Removal with Thresholding Method

The definition of an L-surface, removing from the raw measured data long-components, described as form and waviness, can be supported by the thresholding methods. Generally, the typical thresholding of the surface topography is on its height, considering the segmentation of the analyzed data. However, a simple thresholding method cannot be classified as stable when surfaces have stochastic content. In this case, it can produce many insignificant features, so it can cause problems for many parameters, e.g., the number of defects and the density of features. Consequently, the thresholding method was proposed in many previous studies considering an analysis of the surface topography. Usage of its with wavelet decompositions [100,101] or Wiener filtration [102] was proposed for denoising the roughness measured signal.

The thresholding method was found especially valuable in reducing data processing errors of surfaces containing deep or wide features, such as dimples [103,104]. However, the selection of the thresholded value is another task to be comprehensively studied and adequately resolved. Generally, the application of the thresholding method when analyzing and defining the L-surface is to reduce the influence of the deep/wide features on the position (calculation) of the received plane. It was found in previous studies that size (depth, width) [105,106], density (number) [107], and location [108] (especially edge distribution [109,110,111]) have a considerable influence on the areal form removal process.

**Figure 2:**
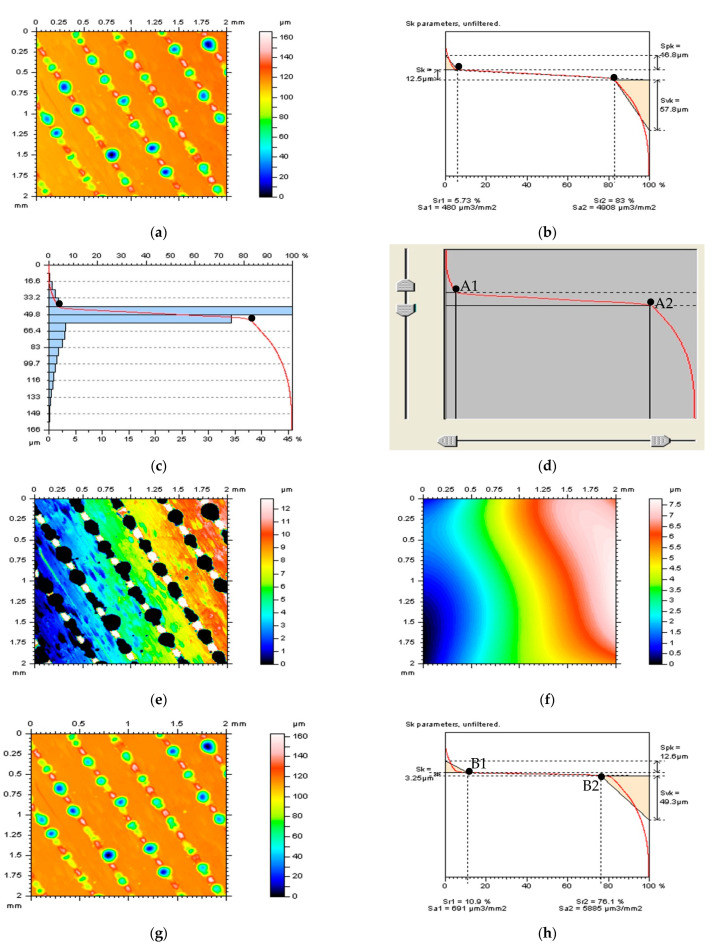
Contour map plots of measured laser-textured surface (**a**), its material ratio (**b**) and Abbott−Firestone curves (**c**), the method of selection of thresholding values A1 and A2 (**d**), the thresholded surface data (**e**), the Poly4 L-surface received from the thresholded surface (**f**), surface received by removal of the Poly4 L-surface from the raw measured data (**g**), and its material ratio curve (**h**).

Figure 2 shows proposals for the selection of thresholding value graphically justified for the laser-textured surface (measured data in Figure 2a). The presented A1 and A2 values (Figure 2d) were located in the areas where the amplitude of changes was the greatest. It can be easily transferred on the Abbott–Firestone (Figure 2c) and material ratio (Figure 2b) curves. The plane corresponding to the L-surface (Figure 2f) can be more easily calculated (with reducing data processing errors) and positioned when the analyzed surface data do not contain deep and wide features (Figure 2e), such as laser-texturing traces. For the received surface after an areal form removal (Figure 2g), the values of the Sk parameter, core roughness depth, calculated as the distance between A1 and A2 values, decreased from 12.5 µm to 3.25 µm (see Figure 2h).

**Figure 3:**
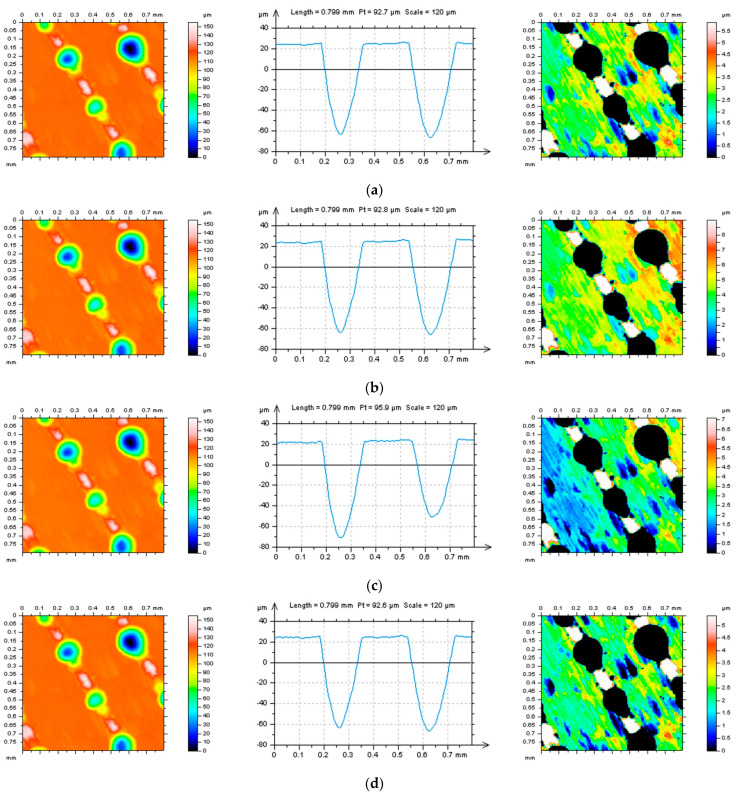

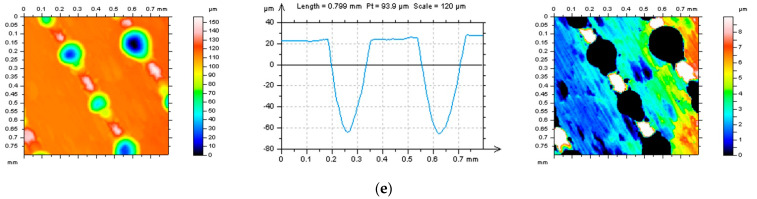
Contour map plots (left column), selected profiles (middle column), and A1−A2 thresholded surface (right column) received from the edge area of laser-textured surface topography after the definition of L−surface by application of Poly2 (**a**), Poly4 (**b**), GRF (**c**), RGRF (**d**), and SF (**e**), cut−off = 0.8 mm.

The thresholding method can also be extremely valuable in validating the areal form removal algorithms. In Figure 3, the thresholding method was applied after this process. Removal of the deep features caused a better recognition of the distortion in the theoretically flat surface. The surfaces after an areal form removal (left column) were thresholded to remove the valleys (middle column), receiving non-dimple data (right column). The more the received L-surface was flat, the better the algorithm for form removal was rated.

#### 2.3.2. Improvement and Validation of Procedures for the Definition and Reduction in High-Frequency Measurement Noise

Considering the improvement in the detection and reduction in high-frequency measurement noise, it was proposed in previous studies to provide multithread analysis. This approach would help in reducing the errors in both processes. Except for the application of ACF and PSD functions, both studies, considering an areal (3D) and profile (2D) [112] characterization, can be valuable. Calculating the ACF and PSD for profiles and surfaces can indicate the occurrence of high-frequency measurement errors.

It was found in previous studies that, in some cases, the profile definition of noise can be more reliable than an areal. In practice, an even surface is characterized by an areal performance, which is crucial in the tribological characteristics of the details properties; extraction of profiles can be essential. Consequently, the identification of high-frequency noise for profile PSD and ACF analysis gave more direct results.

Moreover, the influence of the direction of profile characterization has a considerable impact on the validation of noise removal procedures [19]. The direction has an impact on the results in such a measurement process. For instance, in atomic force microscopy (AFM), the direction parallel to the scanning axis is sampled in less than topography in the perpendicular direction that may take several minutes to measure: the latter is, therefore, much more prone to artifacts from drift. A proposed solution was repeating the measurement on the same surface in three different directions: horizontal, vertical, and oblique. The influence of surface orientation on the variability of measurement results has already been comprehensively studied [113].

**Figure 4:**
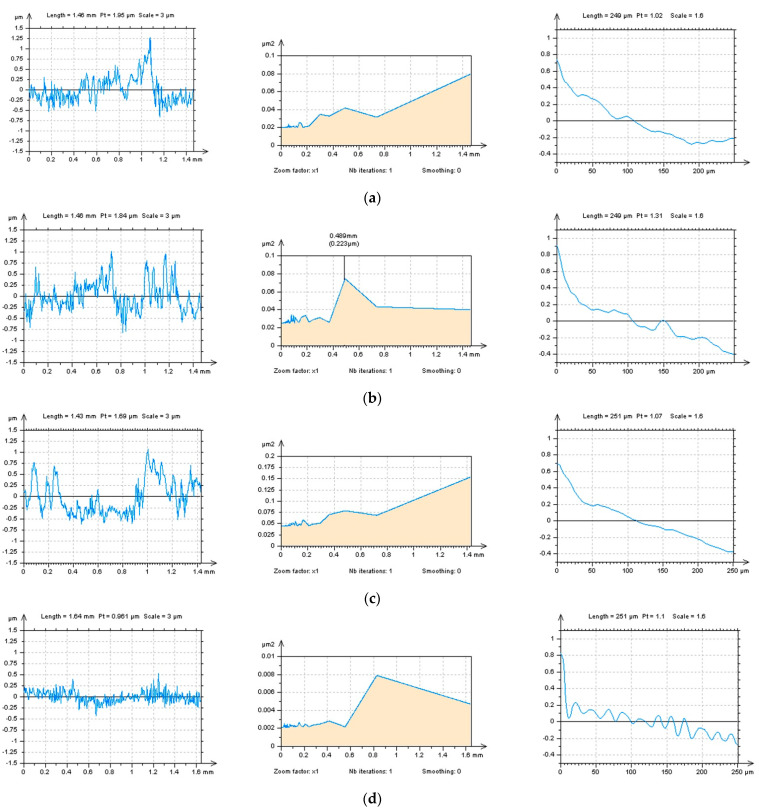

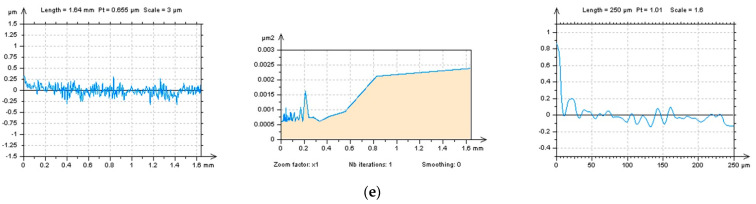
Profiles (left column), their PSD (middle column) and ACF (right column) received from the ground surface by extraction in: horizontal (**a**), vertical (**b**), random (**c**), treatment−trace peak (**d**), and treatment trace valley (**e**) directions.

Figure 4 (left column) presents profiles received by extraction (from the ground surface) in different directions. Except for the traditional horizontal (a), vertical (b), and random (oblique) (c) directions, the treatment trace (d,e) was utilized. This technique can depend on the peak or valley location. The treatment trace peak direction is consistent with the direction of the peak trace on the machined surface, and the treatment trace valley is in line with the direction of the valley trace. It was found that the validation of the treatment trace technique depends on the peak and valley details of the type (plateau-honed, turned, ground, laser-textured, or, generally, textured) of the analyzed surface topography. From the results obtained for the PSD (middle column) and ACF (right column), the horizontal, vertical, and oblique directions did not allow for the detection of high-frequency measurement noise from the results of surface roughness. For the peak characterization (Figure 4d), the PSD did not justify if the noise existed, but respective differences in the shape of ACF could indicate some noise occurrence. Both methods (PSD and ACF) indicated that high-frequency errors can occur in the results of surface roughness measurements when the treatment trace valley direction was selected (Figure 4e). Response from that matter is that, if the amplitude of the surface data is relatively high, the detection of high-frequency measurement errors from the roughness measurement is difficult, even when the multithreaded analysis is provided. It is suggested to define the profile with the lowest height amplitude when detecting the high-frequency measurement noise with the directional extraction method.

**Figure 5:**
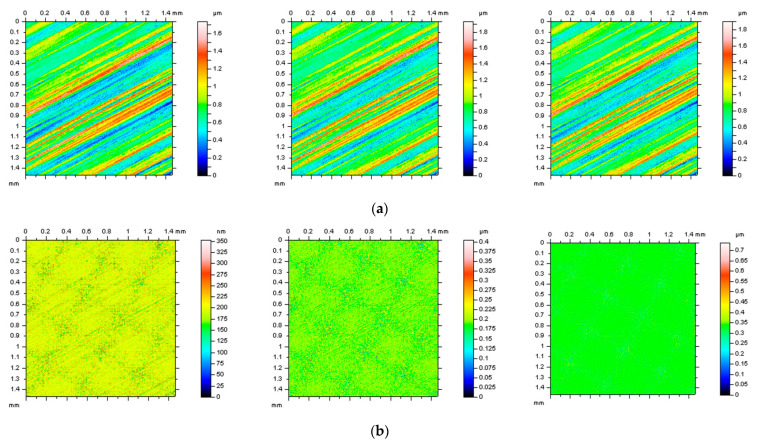

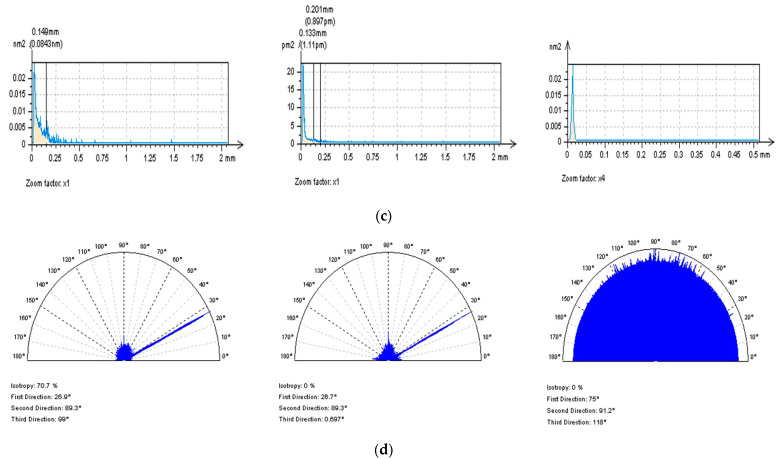
Contour map plots of the ground surface after S-surface removal (**a**), defined S−surface (**b**), PSDs (**c**), and TDs (**d**) of S−surfaces received by application of GRF (left column), SF (middle column), and FFTF (right column), cut−off = 0.10 mm.

The accuracy in the reduction in high-frequency measurement errors can be lost, even if the detection is provided appropriately, i.e., the noise removal procedure (e.g., digital filtering) can increase the distortion of results. From that issue, an analysis of the noise surface (NS) was suggested. The NS is a surface that consists of high-frequency measurement noise. In practice, it is a surface received by S-filtering with an application of the S-operator [44] or, simplifying, S-filter. It was proposed that precisely defined NS should consist of only the high-frequency components and, respectively, should be isotropic. In Figure 5, various NS plots were presented. They were received by various digital filtering methods, especially those commonly used (available in commercial software). Except for the analysis of contour map plots (Figure 5b) of the NS, and similarity in the PSD functions (Figure 5c), the differences indicating the algorithm precision were visible in the texture direction (TD) graphs calculated for the NS (Figure 5d).

## 3. Results

Studies of ST were divided into two subsections. Firstly, in Section 3.1, the errors in the definition of L-surface were presented, and their reduction was proposed. Secondly, in Section 3.2, proposals of procedure for the minimization of errors in the S-surface definition were presented. For both methods, results were validated and presented.

The main course of the studies was to, firstly, select an appropriate method (e.g., degree of least-square fitted polynomial plane) for an areal form removal (definition of the L-surface) and then to identify the digital filter (with cut-off value) causing the smallest errors in processed data (and ISO 25178 parameters). Both operations (definition of L-surface and S-surface) were improved with an application of commonly used (available in commercial software) functions, such as ACF, PSD, and TD graphs.

### 3.1. Reduction in Errors in the Definition of the L-Surface

The definition of an L-surface usually depends on the precision in the minimization of surface topography feature distortion. The exaggregation of features, such as dimples, oil pockets, scratches, or, generally, dimples, increased when they were located near the edge of the analyzed detail. Moreover, when the surface contained deep and wide dimples, distortion increased enormously as well. Some proposals can be found with valley extraction [110]. Not only the application of the too-large degree of the least-square fitted polynomial (LSFP) plane [33] can cause a dimple distortion; digital filtration, such as regular Gaussian regression (GRF), robust Gaussian regression (RGRF), or spline (SF) filters, can grossly distort selected surface topography features. The bi-square modification of polynomials of the nth degree can reduce those errors; nevertheless, their application requires mindful users [114]. Considering the distribution of features, the areas located between deep and wide features and the edge of the analyzed details were also vulnerable to greater distortion, contrary to the areas where such features were not located. This disadvantage was especially visible for digital filtering, even though the bandwidth was enlarged. For some solutions, the cut-off was proposed to be enlarged; nevertheless, on the other hand, it may have caused the form was not removed entirely. An exemplary solution can be found when the features are not distorted, but the out-of-feature [13] surface is completely flat. To receive these data, features must be excluded from the surface. One of the methods for extraction (removal) of features from the raw measured data is an application of the thresholding method, widely presented and proposed in this study.

**Figure 6:**
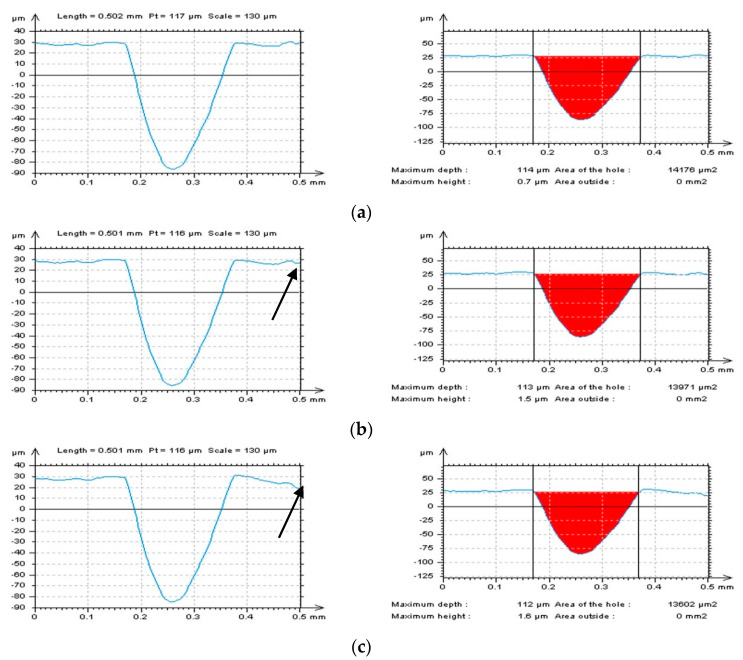

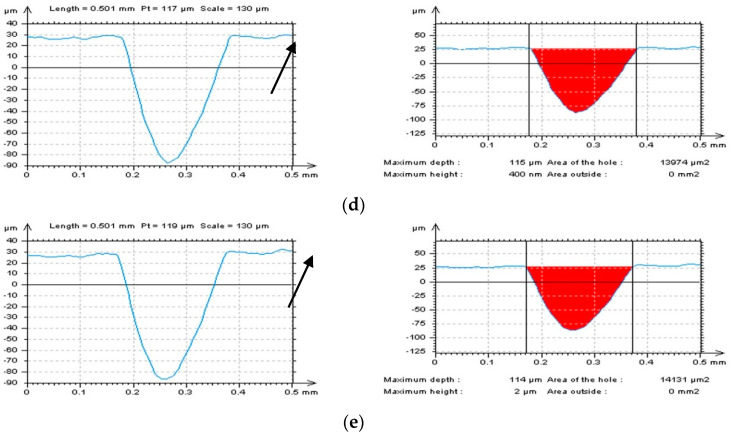
The dimple profiles (left column) and their hole/peak area diagrams (right column) received from the edge area of laser−textured surface topography after the definition of L−surface by Poly2 (**a**), Poly6 (**b**), Poly10 (**c**), GRF (**d**), and SF (**e**), cut−off = 0.8 mm.

In Figure 6, selected profiles and their hole/peak area diagrams were presented. They were received from the surface after an areal form removal by various methods. Increasing the degree of LSFP resulted in distorted edge-located areas where the dimples occurred. It was also found that exaggeration was enlarged when the size (depth and width) of the feature increased. The greater the features, the larger the distortion (Figure 6a–c). Application of GRF and SF seems to be the most encouraging solution; nevertheless, not always the entire form, especially waviness, was eliminated. From that matter, the application of the 2nd degree of LSFP (Poly2) seems to be the most suitable for the definition of the L-surface. However, it must be considered that a low (e.g., second) degree of a polynomial would not entirely remove the form from the measured raw data. Some proposals can be found in the application, firstly the polynomial of the second degree (removal of shape) and then the usage of digital filtering (e.g., GRF or RGRF) to eliminate the waviness. However, increasing the number of methods applied can significantly extend the time of data processing and, unfortunately, enlarge the number of errors in data analysis. It is best to remove the form (shape and waviness) entirely.

**Figure 7:**
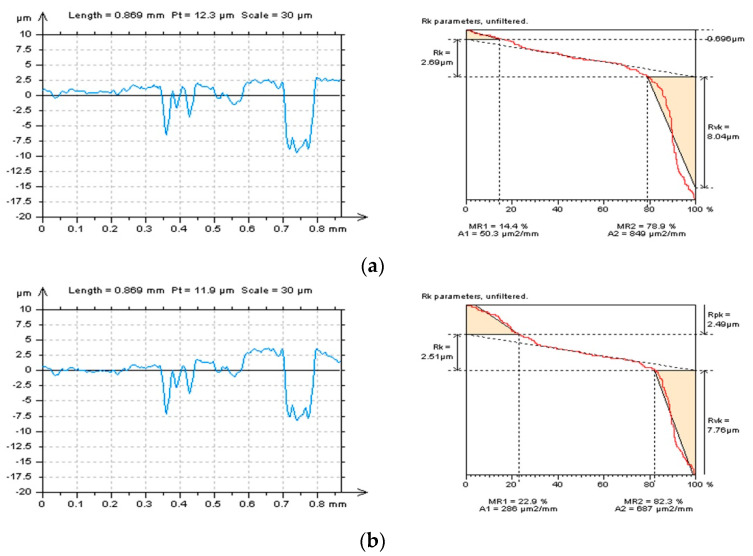

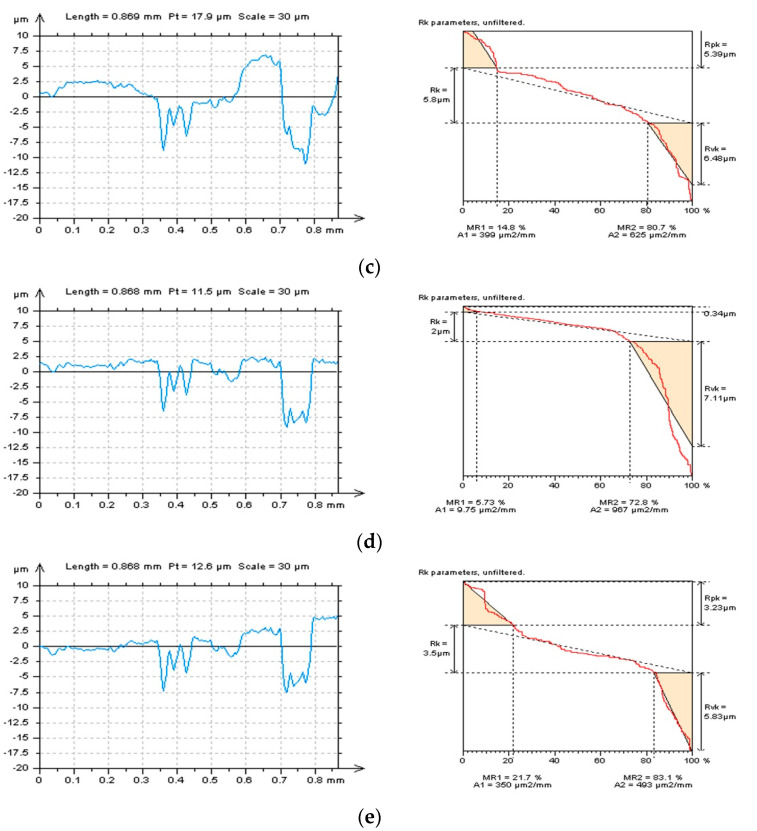
Selected profiles (left column) and their graphical studies of Rk−group parameters (right column) received from the laser−textured surface topography after the definition of L−surface by Poly4 (**a**), Poly8 (**b**), Poly12 (**c**), RGRF (**d**), and SF (**e**), cut−off = 0.8 mm.

Distortion in the features can be particularly noted for the profiles presented in Figure 7 (left column). Except for the exaggregation of features and edge-located areas of details, tilt can also be found, even if the surface was previously leveled [115] (according to the guidance provided by the software). It is another interference with the data and, correspondingly, can enlarge the possibility of data distortion. From all of the methods presented in Figure 7, Poly4 or RGRF seem to give the most encouraging results. However, additional leveling is required. When defining an appropriate L-surface, firstly, the distortion of features is not allowed; secondly, the out-of-feature part of the surface should be flat. The thresholding method can be proposed for both improving the form removal methods and validation of the approach already applied.

### 3.2. Selection of a Method for S-Surface Definition with a Suppression of the High-Frequency Noise

The process of selection of the method for the reduction in a high-frequency measurement noise was proposed with areal (3D), profile (2D), ACF (areal and profile), PSD (2D and 3D), and TD analyses. It was suggested that all of the required properties should be studied with a multithreaded characterization. The measurement noise, similar to the uncertainty [41,116,117], can be reduced by repeating the measurement process of the same probe (detail). However, noise, especially in the high-frequency domain, can be characterized as separated data from those measured raw. The results received after S-filtration were defined as noise surface (NS) [39]. Some significant properties of the NS were defined and analyzed, considering the validation of noise removal methods, such as Gaussian (GRF or RGRF), spline (SF), median denoising (MDF), and fast Fourier transform (FFTF) filters, all available in commercial software.

**Figure 8:**
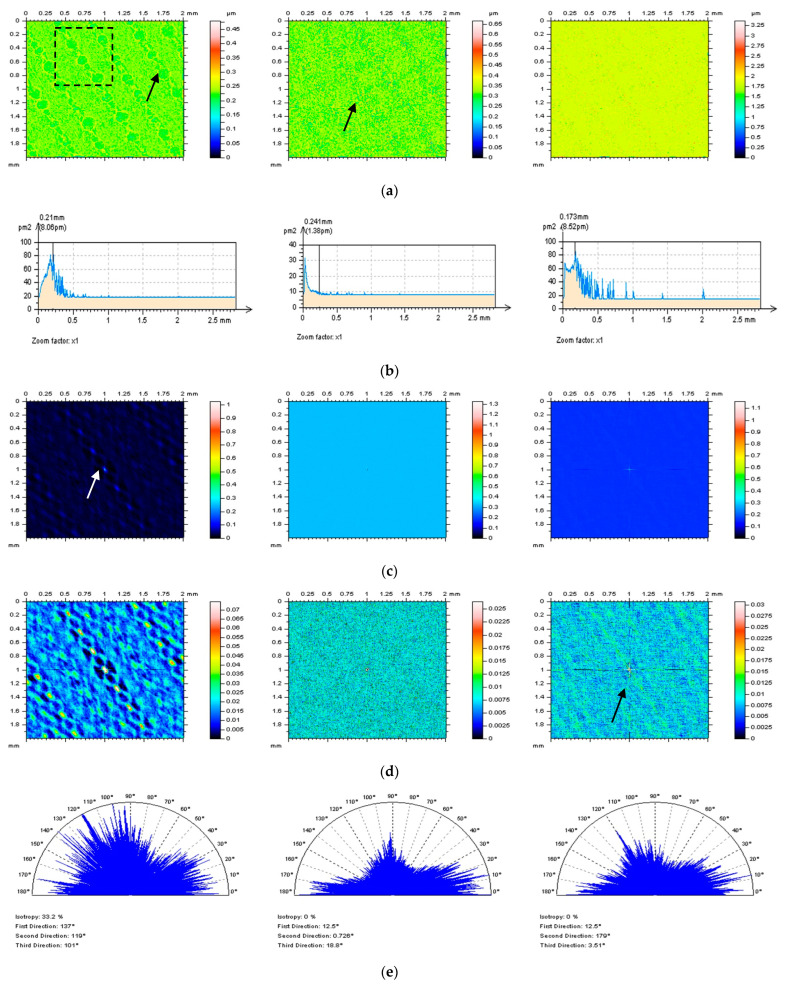
Analysis of NS: contour map plots (**a**), its PSDs (**b**) and ACFs (**c**), thresholded ACFs (**d**) and TDs (**e**) received after application of GRF (left column), SF (middle column), and MDF (right column), cut−off = 0.010 mm; studies provided for the laser-textured detail.

According to the first NS property, it should contain only the required noise frequencies. In the considered case, only high-frequency components must be defined in the NS. Some non-noise features can be received when analyzing the isometric view of the surface. In Figure 8a, three various NS were presented and obtained after the application of the GRF, SF, and MDF methods (cut-off = 0.10 mm), respectively, from left to right. From that analysis, the NS received by GRF and SF filtration included some unwanted elements, indicated by the arrows, and it seems that the MDF is the most encouraging method.

Considering the second NS property, it should be in the domain of noise. In the analyzed example, the NS should be in the high-frequency domain. For validation of this issue, the PSDs (Figure 8b) were considered. From that matter, all of those three filters gave suitable results; nevertheless, NS created by the SF accumulated the most high-frequency components.

As the third issue, the ACF of NS should be isotropic, as the NS itself. In Figure 8c, the 3D ACFs were presented for each of the NS. The GRF method created NS with ACF containing non-noise components. Moreover, the ACF consisted of some non-noise features (identified by the arrows). The ACFs received after SF and MDF filtering seemed not to contain any non-high-frequency-noise components. For the validation of this property, the thresholding method for ACF characterization was proposed (Figure 8d). Application of the thresholding technique, with considerable A1 and A2 values, confirmed non-noise components on the GRF NS, but it also indicated that NS obtained by MDF filtration contained the unwanted elements (presented by the arrows). According to those results, the MDF seems to be unsuitable for the extraction of high-frequency errors from the results of surface topography measurement of laser-textured surfaces.

The isotropic property of NS can be additionally studied with an analysis of TD graphs. Figure 8e presents TD graphs of all the three NSs studied in this work. From that issue, all three compared algorithms (GRF, SF, and MDF) gave no reliable responses. The isotropic property was not received for each of the filters matched. However, if selection must be proposed from those methods, the SF seems to give the most appropriate response (from those analyzed) for the suppression of high-frequency noise from the results of laser-textured surface topography measurements.

**Figure 9:**
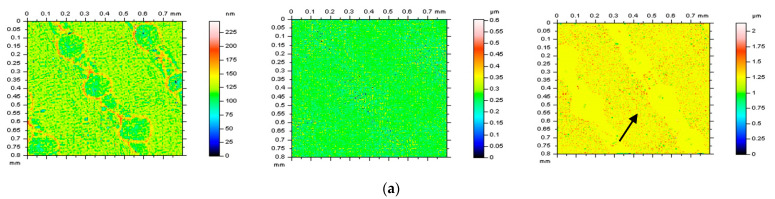

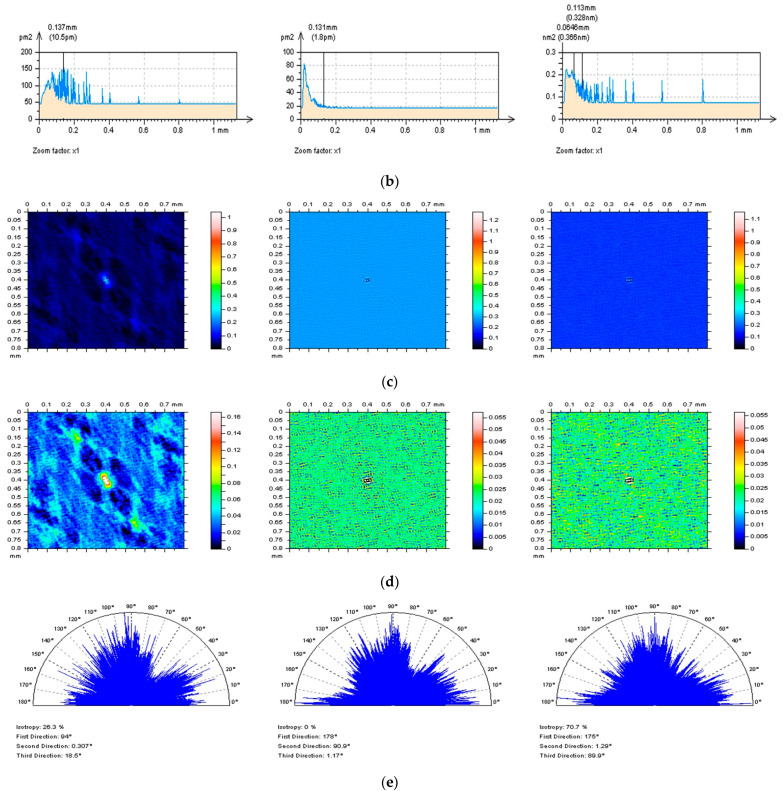
Analysis of extracted details from the NS: contour map plots (**a**), its PSDs (**b**) and ACFs (**c**), thresholded ACFs (**d**) and TDs (**e**) received after application of GRF (left column), SF (middle column) and MDF (right column), cut−off = 0.010 mm; description of the enlarged detail location was presented in Figure 8a (first left).

Some improvements in the validation of the method can be obtained when NS enlarged details are considered. An example of an enlarged detail (0.8 mm × 0.8 mm) was presented in Figure 9. In this case, the isometric views (Figure 9a) indicated that NS received by MDF contained non-noise components (it was indicated by the arrow). Contrary to the analysis of the whole detail (Figure 8), the SF NS did not contain non-noise components; it could be falsely estimated by the eye-view analysis. From this example, the multithreaded studies seem to be more justified. In terms of other properties, the PSD validation of the noise frequency dominance, the exclusion of occurrence of the non-noise feature with ACF and thresholded ACF studies, and the TD graphs analysis gave similar responses to those presented for larger detail. This (enlargement) method can validate the analysis of larger details and help in reducing the errors of the S-surface definition. The selection of an appropriate method for high-frequency noise reduction can be improved.

## 4. The Outlook

Despite the many studies presented, there are still many issues that can be addressed. There are some examples below:

1. The proposal of selection of cut-off (as the 3× sampling interval for a stylus, or 3× spacing for optical methods) must be studied and validated for isotropic surfaces. The validation of this type of topographies can be difficult with the methods proposed;
2. The analysis of some isotropic surfaces not containing some treatment traces and directional studies was not comprehensively analyzed. The treatment trace profile characterization may not respond adequately according to the proposals raised;
3. The definition and selection of the thresholding value must be precise for each of the surface types separately. Surfaces after different types of machining can receive various ranges of thresholding values;
4. Errors received by false estimation of thresholding value were not comprehensively studied in this paper. Distortions for each type of machined surface should be described separately as well.

## 5. Conclusions

From all of the studies presented, the following conclusions can be drawn:

1. The false estimation of L-surface when removing the form (shape and waviness) from the measured data can cause huge errors in the calculation of ISO 25178 surface topography parameters and can be the source of classification of properly made parts as lacks, leading to their rejection;
2. The distortion of L-surface positioning increases when the surface contains some deep or wide features, such as dimples, oil pockets, scratches, and valleys. The exaggeration can increase with the enlargement of the feature sizes, density, and distance from the edge of the analyzed detail. Special care must be taken when such features are edge located;
3. To reduce the errors in the definition of L-surface, the thresholding method is proposed. Contrary to the valley-excluding method, the present analysis is faster and does not require additional digital actions allowing to exclude some errors that can arise when the user does not entirely select the feature detail;
4. When selecting the thresholding value, reference to the material ratio and the Abbott–Firestone curves can be advantageous. The thresholding values received for all three functions should be similar or, correspondingly, the difference must be negligible;
5. When selecting the procedure for the definition of S-surface, functions of power spectral density, autocorrelation, and texture direction seem to be required. They must be supported with a mindful analysis of the isometric view of the noise surface;
6. For the characterization of the noise surface properties, thresholding techniques can be beneficial. Supporting this method with a selection of enlarged details can improve the validation of the approach for high-frequency measurement noise detection and reduction;
7. The thresholding method can be advantageous in the process of selection of cut-off values for both the L-surface and the S-surface definitions. Excluding deep or wide features, e.g., thresholding technique, it can reduce the errors in positioning of the reference plane (L-surface) and improve the processes of detection (definition) and reduction (removal) of the high-frequency noise. Moreover, this approach can be found even more crucial in varying the bandwidth value on the type of analyzed surface (e.g., laser-textured).

## Data Availability

Data sharing is not applicable to this article.

## Parameters and Abbreviations

The following abbreviations (left column) and parameters (right column) are used in the manuscript:

*ACF* autocorrelation function	*Sa* arithmetic mean height, µm
*AFM* Atomic Force Microscopy	*Sal* auto-correlation length, mm
*FFTF* Fast Fourier Transform Filter	*Sbi* surface bearing index
*GRF* Gaussian regression filter	*Sci* core fluid retention index
*L-filter* filter used for definition of the L-surface	*Sdq* root mean square gradient
*L-surface* long-wavelength surface	*Sdr* developed interfacial areal ratio, %
*MDF* median denoising filter	*Sk* core roughness depth, µm
*NS* noise surface	*Sku* kurtosis
*POLY2* least-square polynomial of the 2nd degree	*Smc* inverse areal material ratio, µm
*POLY4* least-square polynomial of the 4th degree	*Smr* areal material ratio, %
*POLY6* least-square polynomial of the 6th degree	*Sp* maximum peak height, µm
*POLY8* least-square polynomial of the 8th degree	*Spc* arithmetic mean peak curvature, 1/mm
*POLY10* least-square polynomial of the 10th degree	*Spd* peak density, 1/mm^2^
*POLY12* least-square polynomial of the 12th degree	*Spk* reduced summit height, µm
*PSD* power spectral density	*Sq* root mean square height, µm
*RGRF* robust Gaussian regression filter	*Sr1* upper bearing area, %
*RMS* root mean square height	*Sr2* lower bearing area, %
*S-filter* removes small-scale lateral components	*Ssk* skewness
*S-L surface* a surface received after S- and L- filtering	*Std* texture direction, °
*S-surface* small-wavelength surface	*Str* texture parameter
*SF* spline filter	*Sxp* extreme peak height, µm
*TD* texture direction (graph)	*Sv* maximum valley depth, µm
	*Svi* valley fluid retention index
	*Svk* reduced valley depth, µm
	*Sz* the maximum height of the surface, µm
